# Supplementary figures and images for: Inhibition of polymerase chain reaction: Pathogen-specific controls are better than human gene amplification
Source: PLoS One. 2019 Sep 27;14(9):e0219276. doi: 10.1371/journal.pone.0219276 (PMC6764677; doi:10.1371/journal.pone.0219276)

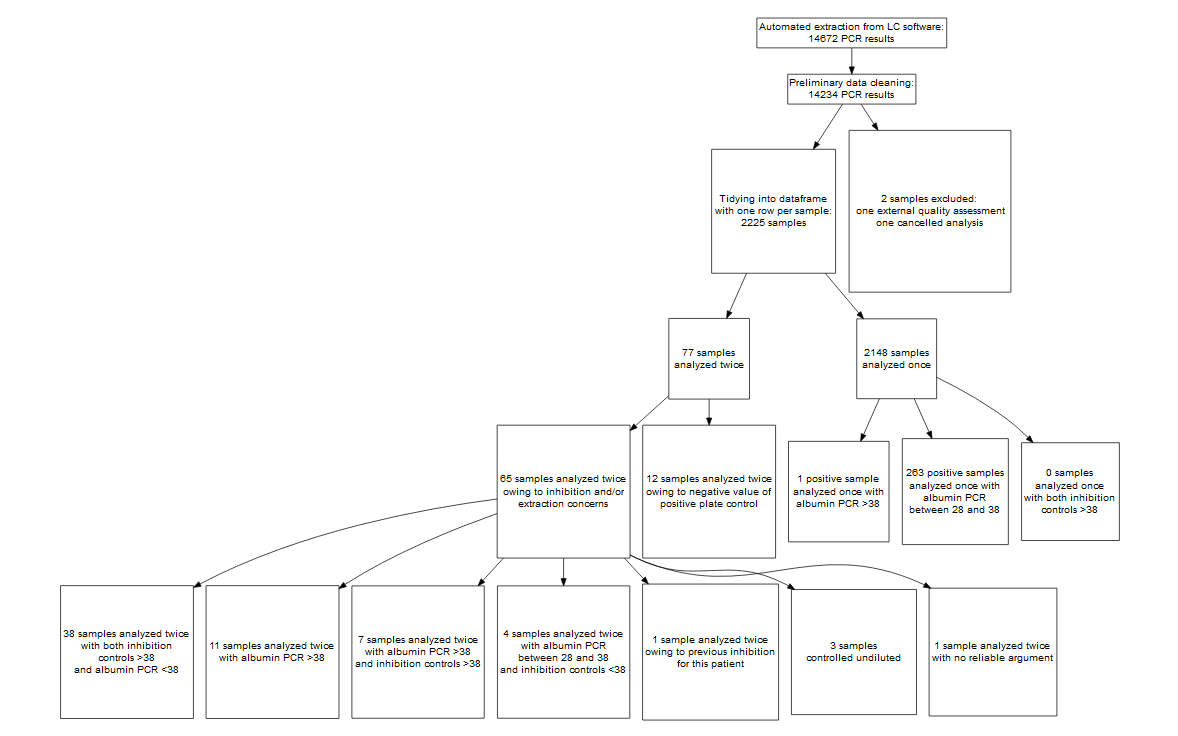


### Figure S1: Flow chart of data cleaning and tidying of *Toxoplasma* PCR results

Supplement: S1 Fig — (DOCX) [file pone.0219276.s002.docx]

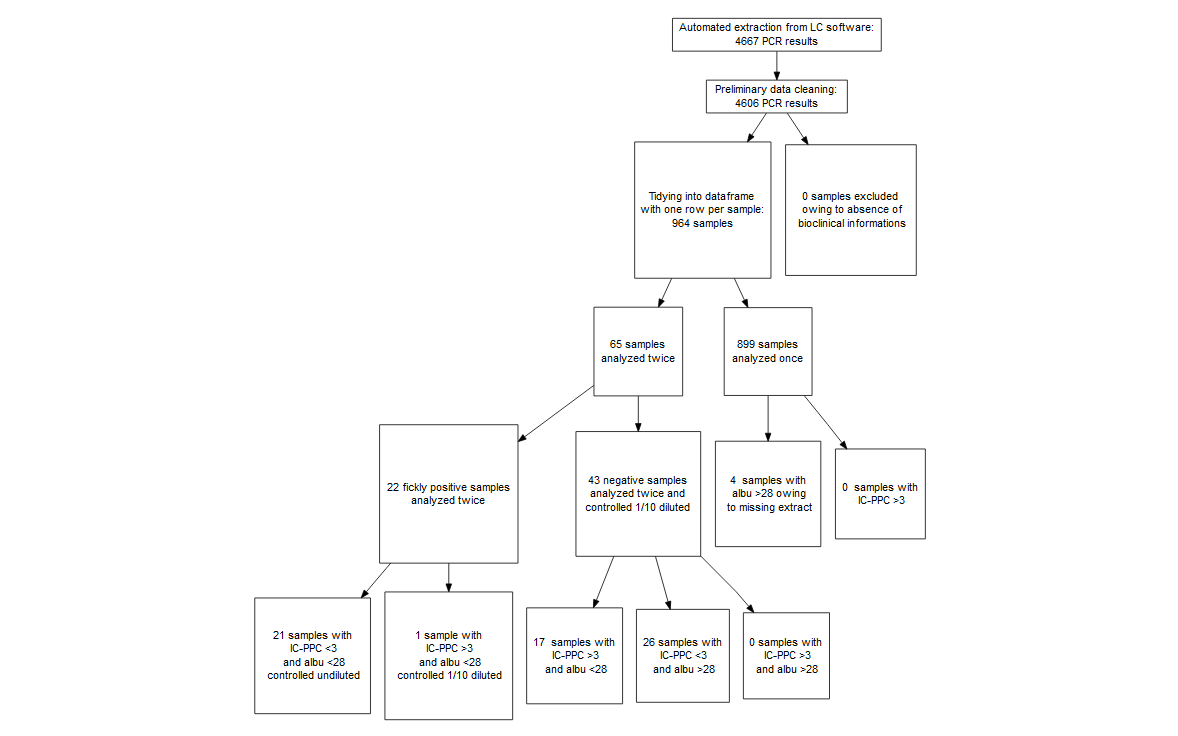


### Figure S2: Flow chart of data cleaning and tidying of *Pneumocystis* PCR results

Supplement: S2 Fig — (DOCX) [file pone.0219276.s003.docx]
