# Supplementary material for: Inhibition of polymerase chain reaction: Pathogen-specific controls are better than human gene amplification
Source: PLoS One. 2019 Sep 27;14(9):e0219276. doi: 10.1371/journal.pone.0219276 (PMC6764677; doi:10.1371/journal.pone.0219276)
Supplement: S3 Fig — (DOCX) [file pone.0219276.s004.docx]

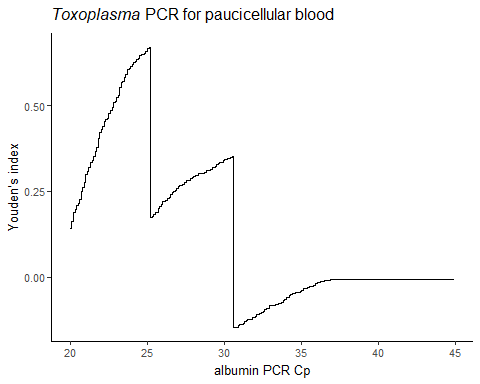

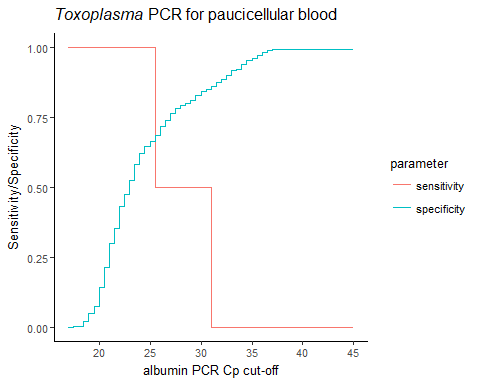

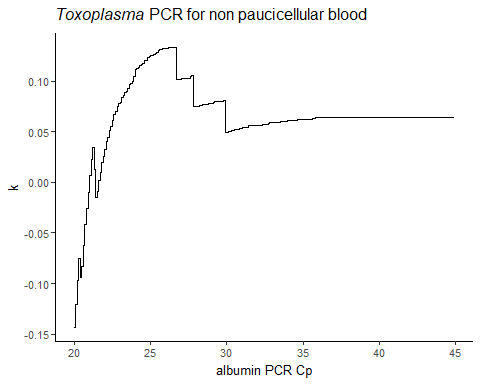

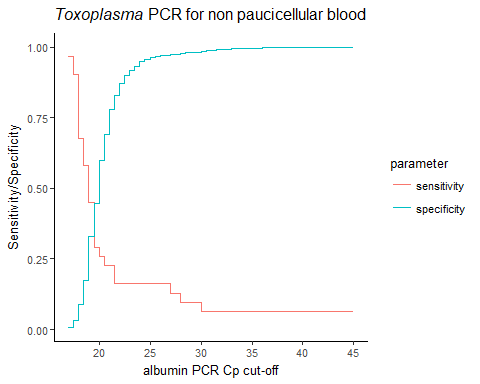

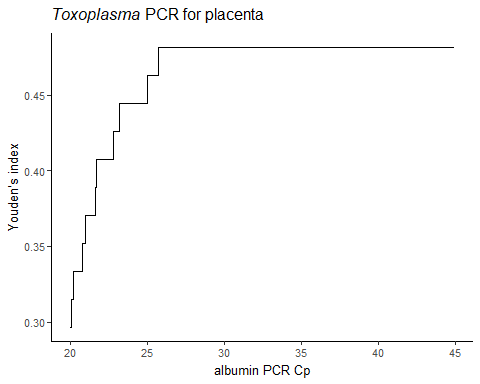

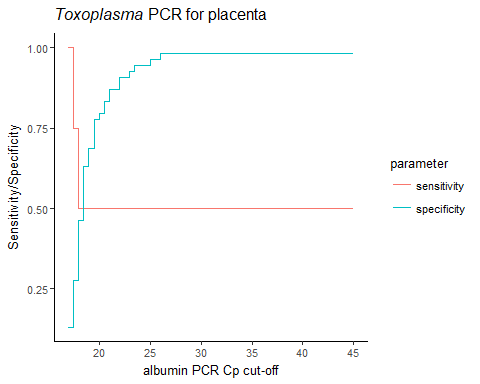

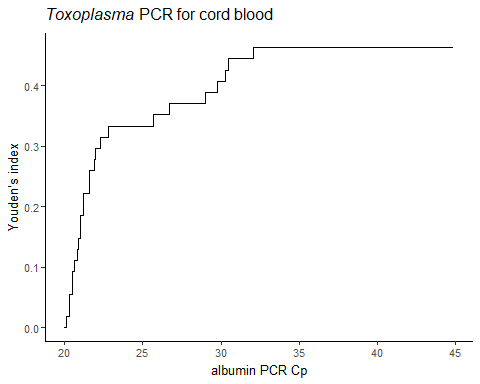

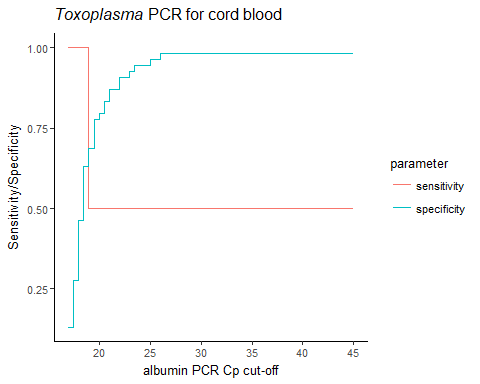

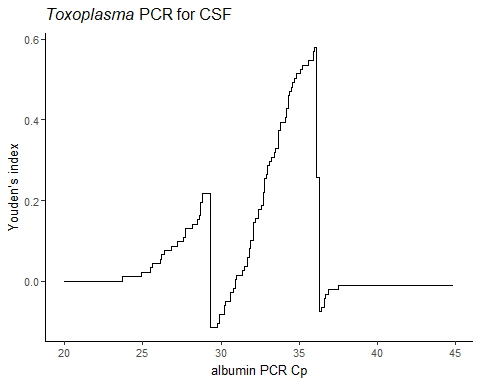

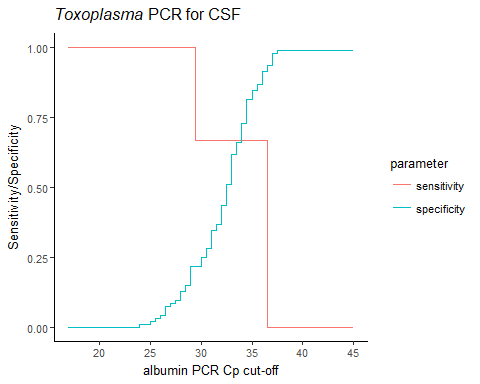


### Figure S3: Youden’s index, sensitivity and specificity of albumin PCR cut-off values for the most frequent matrixes analysed in *Toxoplasma* PCR
