# Supplementary material for: Inhibition of polymerase chain reaction: Pathogen-specific controls are better than human gene amplification
Source: PLoS One. 2019 Sep 27;14(9):e0219276. doi: 10.1371/journal.pone.0219276 (PMC6764677; doi:10.1371/journal.pone.0219276)
Supplement: Attachment — Submitted filename: Roux_Inhibitors_ResponseToReviewers_PLoSOnev190816.docx [file pone.0219276.s006.docx]

PONE-D-19-16663
Original research. Inhibition of Polymerase Chain Reaction: Pathogen-Specific Controls are better than Human Gene Amplification
PLOS ONE

Dear Editor,

Please find a revised version of the manuscript that addresses all the points raised during the review process.

Journal requirements:

When submitting your revision, we need you to address these additional requirements. 1. Please ensure that your manuscript meets PLOS ONE's style requirements, including those for file naming. The PLOS ONE style templates can be found at http://www.journals.plos.org/plosone/s/file?id=wjVg/PLOSOne_formatting_sample_main_body.pdf and http://www.journals.plos.org/plosone/s/file?id=ba62/PLOSOne_formatting_sample_title_authors_affiliations.pdf

**Done**

2. Thank you for stating the following in the Financial Disclosure section:
""RSI Assurance Maladie Professions Libérales - Provinces. C.A.M.P.L.P." has paid the LightCycler 480 (Roche®) real-time PCR equipment in 2009
The funders had no role in study design, data collection and analysis, decision to publish, or preparation of the manuscript."

**Done**

Competing Interests Statement: RSI Assurance Maladie Professions Libérales - Provinces. C.A.M.P.L.P. has paid the LightCycler 480 (Roche®) real-time PCR equipment in 2009; this does not alter our adherence to PLOS ONE policies on sharing data and materials. Please respond by return email with your amended Competing Interests Statement and we will change the online submission form on your behalf.

**Responded by email August the 7th**

Reviewers' comments: Reviewer's Responses to Questions
**Comments to the Author**
3. Have the authors made all data underlying the findings in their manuscript fully available?
Reviewer #1: No

**Right, by mistake, we forgot to upload the spreadsheet of raw data. We have now provided the analysis pipeline on GitHub (Project name “PCR-inhibition” and author “ROUXGUI”).**

5. Review Comments to the Author
Reviewer #1: Authors present their work on inhibition of PCR. I found this work useful. Following are my few suggestions in general and few minor edits for the manuscript, that authors may consider.

Overall, it’ll be a good to provide a brief discussion of the work in the context of it’s utility to other type of pathogens as well.

**Yes, a sentence was added is the discussion**

In addition, it’ll be helpful for others if authors could provide the analysis pipeline on GitHub or something similar. Since they used R, therefore it should be easy.

**Done, see above**

Authors may also improve the quality of figures as I found them bit blurred.

**Done**

Minor comments
Line 140: I think it should be pathogen- specific amplification.

**Yes,** **modified, this typo was present twice in the manuscript (L115 and L140)**

Line 148-150: Is the p-value significant computed after correction or before?

**After the Holm correction. Text has been rephrased in an unambiguous manner**

Line 194: should be pathogen-specific control. Also, please check space issues elsewhere in the article.

**Done, this typo was present three times in the manuscript (L227 and in the caption of Figure 2)**

While revising your submission, please upload your figure files to the Preflight Analysis and Conversion Engine (PACE) digital diagnostic tool, <https://pacev2.apexcovantage.com/>. PACE helps ensure that figures meet PLOS requirements. To use PACE, you must first register as a user. Registration is free. Then, login and navigate to the UPLOAD tab, where you will find detailed instructions on how to use the tool.

**Done**

I hope that this manuscript will now be considered acceptable for publication in PLoS One.

Yours sincerely

Yvon Sterkers
